# 93 Health Department Generated Antibiotic Prescribing Feedback Reports using Commercially Available Data, New York City, 2025

**DOI:** 10.1017/ash.2026.10717

**Published:** 2026-06-23

**Authors:** Lindsay Bow, Jeanne Sheffield, Arthur Vaught, Stephanie Mayoryk, Chiquacta Hodges, Lisa Maragakis

**Affiliations:** 1 The Johns Hopkins Hospital; 2 Johns Hopkins Hospital; 3 Johns Hopkins University School of Medicine

## Abstract

**Background:** Endometritis is one of the most common perinatal complications, affecting 7.0% of cesarean deliveries occurring after the onset of labor and 0.2-2.0% of vaginal deliveries. Chorioamnionitis increases the likelihood of delivery via cesarean section and the risk of subsequent postoperative endometritis. The addition of chorioamnionitis to the 2026 National Healthcare Safety Network (NHSN) definitions for infections present at the time of surgery (PATOS) aligns surveillance methods with a common mechanism for post cesarean endometritis, which NHSN defines as an organ space surgical site infection (SSI). **Methods:** This retrospective, observational study evaluated 6,340 cesarean deliveries at an academic medical center in the United States from January 1, 2019 through September 30, 2025. Cases were identified via electronic chart review using the NHSN criteria for endometritis. **Results:** During the study period, 59 organ space SSIs were identified, accounting for 0.9% of cesarean deliveries. Seventy-three percent of the organ space SSIs met the definition of endometritis (n=41). Chorioamnionitis was suspected in 14.6% (n=6) of the endometritis cases at the time of their cesarean delivery. Among the cases with chorioamnionitis, 66.7% (n=4) had documentation within the operative note that would meet the updated NHSN requirements for PATOS. Other documentation was included in progress notes following the procedure, which would not qualify for PATOS criteria. Subclinical chorioamnionitis was detected on placental pathology in three additional cases. Compared to the average onset for all postoperative endometritis cases (5.3 days), patients with suspected chorioamnionitis had an earlier onset (1.5 days). Over a third (34.1%) of endometritis cases were identified on postoperative day 0 or 1 (n=13). Chorioamnionitis was suspected in 21.4% of these early endometritis cases. **Conclusion:** The addition of chorioamnionitis to the PATOS definitions for surgical site infections reduced cesarean delivery SSI rates by 10.2% at our center and marks an alignment of surveillance and clinical definitions. In adopting this new definition into surveillance practices, obstetric and infection control teams should collaborate to ensure appropriate identification of chorioamnionitis cases, particularly when considering onset of endometritis in the early postoperative period.

